# Contemporary views on the future of physiology—a report from the 2019 P-MIG focus group

**DOI:** 10.3389/fphys.2023.1176146

**Published:** 2023-06-06

**Authors:** Luis Monteiro Rodrigues, João Gregório, Erica Wehrwein

**Affiliations:** ^1^ CBIOS Universidade Lusófona’s Research Centre for Health Sciences and Technologies, Lisboa, Portugal; ^2^ Department of Physiology, Michigan State University, East Lansing, MI, United States; ^3^ P-MIG The Physiology Majors Interest Group, East Lansing, MI, United States

**Keywords:** human physiology, present and future, themes and trends, major concerns, education and research

## Abstract

Physiologists are seen as professionals with a unique understanding of life, health, and disease, essential to the progression of knowledge regarding human functions and health. Among these experts however, the thematic of the “Future of Physiology” has been regularly present in the agenda of physiology organizations around the world as various uncertainties about teaching and research in human physiology emerged. The Physiology Majors Interest Group (P-MIG) 2019 meeting provided the occasion for some strategic reasoning and planning, aiming to identify the trends that might drive future changes in human physiology. Twelve physiologists, all experts in different areas of Physiology research and teaching, nearly all based in North America, volunteered to participate in this focus group. The session was audio recorded. A verbatim transcript of the recording was then analyzed through thematic analysis, aiming to identify the most relevant themes for the future of Physiology and how these themes might unfold. The group concluded that a shared consciousness on general goals is present, meaning to preserve and develop the interdisciplinary/integrative nature, to promote more innovative teaching/learning practices, and to acknowledge technology as the main catalyst for research and teaching innovation and progress. This consciousness was present in all participants. The group also concluded that transformation will likely need to be more effective, and should involve the Physiological Societies and organizations around the world. Special emphasis was placed on the need to share common competences for curriculum definition, common guidance for teaching practice, and common assessment procedures, with particular attention recommended toward science communication.

## 1 Introduction

Physiology is regarded as vital in all biomedical fields being currently an essential component of many health sciences courses. Nevertheless, some alarming views on a potential decline of physiology evoked justified concerns about how teaching-learning and research might be affected in the coming years (Breckler et al., 2009; Naftalin, 2011; Barman et al., 2013; Gregório et al., 2014; IUPS, 2016). The “Future of Physiology” has been addressed by reference science organizations in recent years (Eisner et al., 2013; IUPS, 2016; Hesketh & Viggars, 2019), apparently rooted in the alleged loss of importance of physiology, and not only in some countries where practical physiology training has nearly disappeared (Naftalin, 2011). The notoriety of Physiology has been progressively blurred by more applied and trendy themes (IUPS, 2016; Gregorio, 2019) while research has become progressively absent in high-rank journals, even if physiology-related. These impacts early career students, scholars, funding agencies, and scientific editors and are noticed in the general public culture as likely some loss of character and identity.

This report results from a focus group organized within the Physiology Majors Interest Group (P-MIG) (www.physiologymajors.org). Although principally based in North America, P-MIG conferences involve experienced international physiologists. The motivation behind this focus group was to better understand current concerns in order to identify major trends that might shape the future of Physiology for the 2030 horizon. An additional goal was to contribute to the development of reliable strategic scenarios for physiological teaching practices, learning, and innovation for the future.

## 2 Methods

This workshop, titled “Future of Physiology,” was held in 2019 during the 4th meeting of P-MIG in Minneapolis, Minnesota (United States).

A literature review on market trends, and other relevant themes was completed by the authors prior to the group assembly. This review allowed authors to become familiar with the latest developments and discussions on this topic and to build an interview script to guide the focus group (Table 1). Alongside these questions, probe questions were used to trigger open discussions with greater depth while helping the moderator to guide the meeting without allowing it to escape to borderline secondary matters.

**TABLE 1:** Final interview script and related probe questions.

1. Do you think Physiology will “exist” within health care education as today, as an independent knowledge domain essential for health professions?
2. How do you think new technological developments (e.g., MOOC’s, online courses, YouTube, Artificial Intelligence, Big Data, Machine Learning) will influence the existence of Physiology and its development?
3. Thinking back, what have been past sources of uncertainty in this field?
4. What are your thoughts about the major stakeholders that could shape or influence research, education and health care?
a. Thoughts on “The Quantified Self” movement, fueled by personal apps like Fitbit (and other fitness apps)
b. “Over demanding” ethical regulations
c. Pressure from anti-animal experiments groups
d. Political orientation of human research (e.g., more applied research vs. fundamental research)
e. Public perception of science
f. Can industry act as “push” or a “pull” factor for Physiology? Is Pharma different from Physiology industry?
5. Think back again about the role of teaching and researching of Physiology in medicine and health sciences. How was your experience 10 years ago? (and before that, when applicable)
6. How to characterize a successful/productive physiologist 10 years from now?
a. How different you imagine it when you think about the last 10 years?
7. What are the major strengths and opportunities that you foresee for Physiology?
8. Do you think different teaching models/methods (especially US and EU) might influence Physiology progression? One way or the other ?

Twelve meeting participants (*n* = 6 males, *n* = 6 females) volunteered for this workshop. These participants were established academic physiologists. All held Ph.D.s.Three participants had 10–15 years of professional experience and nine had more than 15 years experience in this field. Nearly all showed a dual track career involving both research and teaching. One participant had an exclusive teaching track. The majority of participants were from the USA, with two international faculty members involved. All had some involvement at the undergraduate level, eight of them with roles in post-graduate education, and one represented professional school education. A range of subspecialties were included as shown in Table 2. One of the authors, a European physiologist co-writing the script, conducted the session.

**TABLE 2:** Participant characterization according to experience in research, teaching and mentoring in physiology.

Code	Gender	Current location	Education background	Profession	Type institution (U undergraduate; PG posgraduate; P professional)	Main activity in physiology (teaching; research; other)	Subspeciality	Years of teaching experience in physiology (<10; 10–15; >15)	Active mentoring
Moderator	Male	EU	PhD	Professor of Physiology and Pathophysiology	U; PG	Teaching and research	Cardio-circulatory physiology	>15	*n.a*
#a1	Male	United States	PhD	Professor of Integrative Physiology	U; PG	Teaching and research	Renal and electrolyte homeostasis	>15	*n.a*
#a2	Female	United States	PhD	Professor of Biology; Director of Mathematical Biology	U	Teaching and research	Biological membranes and cell adaptation	>15	Yes
#a3	Male	United States	PhD	Professor of Natural Science and Nursing	U; P	Teaching and research	Skeletal Muscle	>15	Yes
						Now only teaching			
#a4	Female	United States	PhD	Professor, Department of Integrative Biology and Physiology	U; PG	Teaching and research	Chronobiology	>15	Yes
#a5	Female	United States	PhD	Associate Professor of Instruction	U	Teaching and research	Human Physiology, Exercise Physiology	>15	*n.a*
				Director of Human Physiology Undergraduate Studies					
#a6	Male	United States	PhD	Professor of Human Physiology	U; PG	Teaching and research	Exercise and environmental physiology	>15	Yes
#a7	Male	United States	PhD	Education Consultant, Former Professor & Chair of Biology	U	Teaching and research	Endocrinology and metabolism	10–15	Yes
#a8	Female	United States	PhD	Professor of Biology	U	Teaching and research in education	Biology education research	>15	Yes
#c9	Female	Canada	PhD	Professor of Physiology	U; PG	Teaching	Scholarship of teaching and learning	>15	Yes
#a10	Female	United States	PhD	Professor, Ecology & Evolutionary Biology	U; PG	Teaching and research	Marine Biology	10–15	Yes
#a11	Female	United States	PhD	Professor of Biology	U; PG	Teaching and research	Bone	10–15	*n.a*
#a12	Male	United States	PhD	Professor of Biology; Physiology Program Director	U; PG	Teaching and research	Cardiovascular and microvascular physiology	>15	Yes

Abbreviations: n. a—information not available.

All participants gave their verbal informed consent to an audio recording of the session for future transcription for thematic analysis. The transcription was imported to MAXQDA v.18.2.0 for Windows (VERBI Software GmbH, Berlin, Germany) to be coded. The codes were then grouped into keywords aiming to relate stakeholders and the identified trends.

The protocol was previously reviewed and approved by the institutional Lusófona University’s Health School (ECTS) Ethics Committee (ref: EC-ECTS/P01.19).

## 3 Results

The session had a total duration of 90 min, including more general ice-breaking questions and personal presentations to start the discussion.

The importance of each theme was discussed following the script outline. After transcription, major themes were arranged around the major stakeholders identified during the session. Among these were, Physiology professionals (primarily teachers and researchers, some entrepreneurs); students; professional organizations and scientific societies; health professions; industry; funding agencies; and science communication and public perception. A detailed description of this analysis is presented in Supplementary Table S1 as supplementary material. A summary of the ideas discussed follows:

### 3.1 Physiologists

Participants recognized Physiology as a discipline with soft borders, permeable to (i.e., benefiting from) the influence of many other disciplines. The capacity for integrating different levels of knowledge, from large (ecological) to small (genetics and molecular biology) was and will continue to be a key skill:

“(what will take to be a successful physiologist ten years from now?) I would say knowledge and flexibility with molecular and genetic techniques is just becoming more and more required.” (participant #a1).

As others noted, physiologists must be comfortable moving through different levels of scientific knowledge. Being able to adapt to new technologies, obtain, and analyze new data will be a relevant part of that integrative ability.

“And then there is us. We have to go through all the levels. It’s like playing chess at three levels; you don’t get to stay at step 1.(…) It’s a cell, in an organ, in an organism, so how does it all work?" (participant #a8).

“…the bridging between those different levels is going to be important. That will remain the goal of the physiologist.” (participant #a4).

“(…) being able to deal with the massive amounts of data that are coming out from these different systems, rather than being able to actually execute one particular technique because that technique probably is going to be automated.” (participant #a6).

### 3.2 Funding

The role of funding agencies, either National Institutes for Health (NIH) or funding from different departments, was considered essential to attract more young people to careers. Participants agreed that funding reductions in related domains has been a consistent problem for some time:

“We were not as attractive to the funding agencies as finding genes even if they were orphans, because that was hot!” (participant #a1).

An immediate effect is the reduction of human resources renewal in both research and education, ultimately affecting the career perception of physiology as a career. To improve their chances of accessing funds, professionals have been looking for partnerships with industry or collaborations with other institutions. In line with this trend, focus group participants considered that diversification of funding sources will be a need more than a trend:

“…in addition to collaborative projects between the institution and industry partners, or work for hire for industry partners. So, I’d say it’s not the lion’s share of what we’ll do but is surely a nice size chunk that helps to keep things going." (participant #a6).

### 3.3 Education

One participant described the “pendulum movement” between professionals who teach physiology courses (physiologists vs. clinicians vs. biophysicists vs. molecular biologists vs. others), certainly changing the way that physiology has been taught.

It was noted that the role of future professionals is to develop “physiological intuition” in students. This physiological intuition implies that the student must develop critical and systemic thinking skills.

“To be successful graduate students, they’re going to have to let go of the idea that they think what they learn is how to do something in my lab. It’s not gonna (sic) count anymore. What they should have learned in my lab was how to ask a question.” (participant #c9).

### 3.4 Technology

Technology is already playing an important part in changing physiologists’ roles in teaching. Recorded experiments can make it easier for students to gain specific knowledge, avoiding searching among materials of unknown sources and quality. Moreover, most students are working toward a diploma at the end of the course, which the emerging and abundant online courses still fail to attribute.

Admittedly, students will look for information on the Internet. As not all information is adequate here, the Physiologist would serve in a coaching role.

“… even better than pre-recorded lectures are short videos. If you take a look at what students are using, five minute YouTube clips, is probably the number one thing … ” (participant #a1).

“(…) taking advantage of these tools, they are out there! And also reinforcing the value of higher education, is that we work through and have you grapple with the information, struggle with it and develop that critical thinking skill versus seeing and watching a bunch of videos, [where] you don’t get that." (participant #a6).

The use of simulators is also expected to increase, as they have improved and become more budget-friendly. The use of simulators has been key to avoid the use of experimental animals. However, a simulator can still be a black box—a physiologist needs to be able to see what is going on inside to understand and decide how it will be used.

“…I get chided by my molecular folks by saying “You are out of touch, because you are actually doing experimental model on an animal, measuring pressure … ” My answer was “when you find me the gene that makes urine, talk to me." (participant #a1).

“Simulators will have to be attractive. Students are used to complex and visually developed computer games. Low quality simulators can easily be disregarded”. (participant #a10).”

“… I worry that if the student goes in and just sees this simulator or whatever it is and it’s a bunch of knobs (…) it may just bore the heck out of them, but is an answer to the issue of animal use." (participant #c9).

### 3.5 Collaboration

Collaboration between physiologists in education and research is clearly visible within the United States. Participants agree that there seems to be reduced hindrance (both institutional and professional) in the US to reaching out to those who may be interested in/helpful to your research. As stated:

“…there are minimal barriers and there’s so much to be gained by having collaborations … ” (participant #a6).

Growing collaboration with industry (the biomedical sensors industry, for example) can be an important factor. Industry might recognize the role of the physiologist approach to develop/test better medical devices. This, in turn, would yield another form of funding. Post-degree learning might also contribute to enhance this collaborative environment between research labs and industry.

The involvement of clinicians in physiology classes was another identified trend. Participants recognized that this option clearly facilitates the applicability character of physiology, not only for the future physician.

“(…) we are all moving towards more case-based teaching (…) As a physiologist, I am not giving control, I am actually collaborating with clinical colleagues in ways we haven’t done before. And so, that’s part of our transition, from ownership “we own this” to, “we are a partnership.” (participant #a1).

### 3.6 Public opinion

Participants recognized the necessity of appropriate science communication, as well as the wisdom and potential benefits of making science relevant to a more general audience.

Communication with the media has suffered recently, with incorrect and misinterpreted publications. However, communication with the public can bypass the media by using new communication channels (e.g., video spots on YouTube; social networking) to pass scientific content within the area. As educators and beyond, one of the best drivers for growing (positive) awareness/increasing the appeal of physiology can be having passionate people talk about what they do—and why they are passionate about it.

“(…) the greatest thing about getting people in the field is getting passionate individuals to talk about the field, why do they do what they do. And film these 30 sec spots, what do I do, why am I excited about? As people are enthusiastic, that excites students.” (participant #a1).

“There’s definitely been a rise in science communication conferences, departments hiring science communication experts. We have a science communication class now that we encourage our students to take. We do workshops with our students on how to make a web page, what to do with a Twitter account, how to use it responsibly as scientists, how to give an elevator talk to non-scientists” (participant #c9).

## 4 Discussion

Strategic planning for organizations or disciplines is a challenging endeavour (Bryson, 1988; Mallon, 2019). Scenarios’ development has been shown to be useful in a wide variety of settings and to yield better results when uncertainties are high (Awasthi et al., 2005; Erdmann and Schirrmeister, 2016; Godet, 2000; Gregorio, 2019; Leufkens et al., 1997; Nørgaard et al., 2001; Schoemaker, 1995). As a part of a scenarios’ exercise, there is a need to identify the underlying trends that will drive future changes in the issue to be studied. This focus group was prompted with that view to contribute to a future scenarios exercise, by identifying the underlying trends, in the industry and market, in regulation and politics. The approach reported here was not meant to predict the future, but rather to better understand the current environment of Physiology within North America (Scearce & Fulton, 2004). The focus group script was developed by the authors and conducted by an experienced physiologist member of P-MIG.

Most relevant findings in this focus group were as follows:

- Interdisciplinary and Integrative Physiology

Underlining that Physiology should keep its experimental character, participants clearly pointed out the interdisciplinary/integrative tendency of modern Physiology as a major movement. The integration of new knowledge and skills and the adaptation to new technologies seems to be as important as the study of bodily functions such as Homeostasis, or Stress Adaptation and Functional Maintenance. More than gaining additional competences, physiologists will need to expand their collaborative network—it is currently common to observe clinical specialists introducing disease mechanisms in physiology. Many Physiology Departments are headed by clinicians with physiology-based experience. This also allows a rethinking of the structure—“joining” departments/labs (Biology, Anatomy, Physiology) might strengthen resources, competences, and teaching practices (evidence-based teaching is clearly another trend, in-line with the STEM paradigm, a conceptual reference for science and technology teaching practices, learning, and research) (Breckler et al., 2009; IUPS, 2016; Scriven & Paul, 1987).

Collaboration between physiologists within the United States likely results from a commonly assumed personality seen in the numerous related associations, nationwide, involving (physiology) departments that preserved their original denomination/character. This same reality exists in many countries from Commonwealth, developed closely to the American STEM system. However, in other countries (e.g., within the European Union), a common framework for teaching physiology does not exist, although some recent programs (e.g., ERASMUS MUNDUS) are providing new initiatives resulting in joint masters degrees also including Physiology (MEME, 2019).

- Teachers, Students and Teaching/Learning Practices

A major challenge regarding high-level teaching is the necessary switch from a content provider to a learning facilitator and promotor of critical thinking, in line with the STEM career goals. This evolution, alongside the multidisciplinary/integrative tendency previously indicated, will take some time to be assimilated by both students and professionals, since the best way to coordinate this process is not clear how (Steury et al., 2015).

Student diversity (e.g., ethnical minorities, new generations from former emigrants), another consequence of globalization, is particularly expressive in the USA as in the EU (Engberg, 2004; Gunn et al., 2015). Student diversity may demand new adaptations to the teaching practices considering the diverse backgrounds present in modern societies (Liu et al., 2010). Nevertheless, e-learning/b-learning are already seen as a good complement to the classroom/laboratory work.

- Technology

Technology is the final catalyst mentioned to ensure high-end reasoning and innovation potential for the future physiologist. New active learning tools such as simulators, video-demonstrations, interactive materials (smart games) are positively regarded as facilitators (Breckler et al., 2009). Technology-based tools will be indispensable for the teaching–learning process and the transformation involved. The emergence of “Big Data” might be one of the driving forces pushing the integrative need for physiology. Better simulators, “wearables” and deeper Artificial Intelligence (AI) are expected to play an important part, especially concerning the experimental component of any physiologist. Simultaneously, these non-pharma industries might be an interesting partner for funding purposes.

The Future of Physiology implies, in essence, the definition of Physiology goals for the foreseeable future. This is an educational challenge that must take into account the identification of necessary skills and must be able to articulate and harmonize its various components. This has been done with the STEM careers in the US, and Physiology is no exception. Physiology’s “resilience” is remarkable, being currently a recognized (knowledge) structure-building undergraduate course in theUnited States, United Kingdom, and other Anglo-Saxon countries (Wehrwein, 2016; Carroll et al., 2017; VanRyn et al., 2017). New teaching/learning practices involving critical reasoning, creativity, and active learning strategies are already in place, with different expressions and frames of reference, focusing skills on cognitive, interpersonal, and intrapersonal dimensions (Andrews & Higson, 2008; Heckman & Kautz, 2012). However, it is not clear how these orchestrate with general and specific goals defined for the future. Complementary efforts from many organizations are noteworthy, contributing to better define professional skills for the future physiologist (French et al., 2020), while promoting the development of this conceptual framework called “core concept” for the Physiology project (Breckler et al., 2009; Crowther, 2017; Gunn et al., 2015; IUPS, 2016; VanRyn et al., 2017). Nevertheless, that articulation is still missing.

The utilization of a focus group of expert physiologists to collaboratively think, discuss, and consider the future of the discipline is the main strength of this study. To our knowledge, a similar exercise has not been presented in the literature thus far, while there are many similar studies about other professionals (e.g., nurses, pharmacists, physicians, other health professionals). Moreover, results presented here might help design prospective scenarios that can be used by research organizations to anticipate future directions in Physiology teaching and research. The main limitation of this study are the limitations of the method—due to the nature of sample, these results may differ with other participants, and cannot be extrapolated for other contexts (e.g., other disciplines). The convenience sample and auto-selection of individuals bring with them some bias, since these participants may have been more interested in participating and more prone to share their vision, which may not reflect the reality of the profession and discipline everywhere. However, we are confident that this bias is not relevant for the main purposes of this study, as the participants were experienced physiologists with a significant number of years in the profession.

## 5 Conclusion

These views indicate that physiologists share common preoccupations. General goals, innovative practices, and global awareness are already installed, although asymmetrically and more randomly than needed. According with these ideas, transformation should be supported on four strategic vectors:

- Common competences for curriculum definition

Specific research in physiology education needs to better articulate the general principles of STEM careers with the identified skills. Those competences should foster a research-validated curriculum template where core (mandatory) disciplines and their relative representation might be compared. Clearly, the integrative component of the program is a highly recommendable direction.

- Common guidance for teaching practice

Science-based interactive learning practices are universally endorsed. Operational definitions of goals for teaching and learning are required and must be clear for teachers.

- Common Assessment

Effective assessment instruments are required to follow-up the competence acquisition process along the way.

- Communication of science

Public awareness is a critical step to regain social recognition and investment.

The authors believe that professional physiology organizations such as the American Physiological Society (US), The Physiological Society (UK) also IUPS (the International Union of Physiological Sciences) or FEPS (Federation of European Physiological Societies) should advocate the necessary changes with the educational policymakers. The design of strategic scenarios supported by the trends revealed in this paper might provide support to develop the most appropriate strategy for each specific context.

Noteworthy, the present reality assembled before the global pandemic forced dramatic changes in our routines, with a special expression in education. It has yet to be determined to what extent these recent changes will alter the path of physiology education. We do feel Physiology is on the right track. However, under this comprehensive framework, more steps are needed to build up a stronger identity, easily recognizable, and able to expand cooperation and career opportunities for the future physiologist—a professional with an exclusive understanding of life, health, and disease, therefore essential to the progression of knowledge regarding human functions in health and disease.

## Data Availability

The raw data supporting the conclusion of this article will be made available by the authors, without undue reservation.
