# Supplementary material for: Contemporary views on the future of physiology—a report from the 2019 P-MIG focus group
Source: Front Physiol. 2023 Jun 6;14:1176146. doi: 10.3389/fphys.2023.1176146 (PMC10279848; doi:10.3389/fphys.2023.1176146)
Supplement: Supplementary file 1 [file Table1.DOCX]

**SUPPLEMENTARY MATERIAL**
**Table SUPLTAB1. Detailed description of the main themes identified in the focus group**

| **Theme** | **Description** |
| --- | --- |
| **Main stakeholders** | - The main stakeholders identified were professionals, major undergrad and graduate students, professional organizations, scientific societies (e.g., APS), schools and colleges, other health professions, funding agencies (NIH, federal funding – Defense and Naval departments) and industry (devices-tech, pharma) and public perception. |
| **Funding** | - Funding is estimated to be low compared to applied sciences. The integration of Physiology with other sciences was seen as the factor that triggered the loss of funding identified since the 1960s/1970s. - Funding Physiology allows the development of research projects, but it also attracts more young people into careers. The referred funding difficulties reduced job opportunities in research, also impacting education. - Professionals have been looking for alternative forms of financing, either by partnerships with industry or collaborations with other institutions to improve the chances of accessing money. There is a feeling that there is pressure to publish and that the increase in publications allows access to more funding. |
| **Health professions** | - Understanding the functioning of a healthy body is essential for all health professions. Access to health courses in the USA is done through pre-graduate programs. There was a time when cellular and molecular biology gained dominance in pre-graduate programs; Currently, an increasing demand for undergraduate Physiology programs is apparent, and this was identified as a trend that will remain in the future. - The proximity to health professions also promotes collaboration with clinicians to participate in teaching activities. This may provide focus on health themes such as hypertension, diabetes, and clinical (physiological) research, more relevance for clinical practice and public awareness. |
| **Physiology Professionals** | - University seniors currently finishing their majors seem to find jobs quickly. - Access to the profession through research is increasingly difficult. Experts agreed that 1/12 of graduated students will aim to reach a “tenure position”. - In the USA, a “hibernation” effect might be seen in the development of the profession. The loss of importance in graduate courses is curbed by the emergence in pre-graduate courses. But only when the current graduates of pre-graduate courses reach positions in the academy that allow them to develop their work will the profession return to the fore. - Educational competence is a growing requirement. Traditional physiologists will have difficulties with this change because they have not been trained or do not have the skills to make that shift. - Physiologists must be trained to cooperate with other professionals from different fields (e.g., cell and molecular biology). |
| **Professionals - Role in teaching** | - Information abounds and it is necessary to develop skills in students that allow them to navigate that information, to know how to identify sources, and apply the collected knowledge. Teaching will no longer have an “apprentice” matrix, based on a direct student-master relationship (where it would be expected that the student would later assume the role of the master). The focus lies mostly in preparing students for the job market in the industry. - The role of today's professionals seems to be the development of “physiological intuition” in students which is also an active instrument of self-learning. The physiologist will act as a mentor, leading the student to develop critical thinking and systemic thinking skills. The mentor's goal is to promote advanced reasoning. The aim is to add significant value to education. - To master communication technologies seems to be crucial since experiments, demos and specific themes can be easily produced and distributed to ease up students work. It is certain that students will look for information on the internet, where information is frequently not previously screened. The coaching role of trained Physiologists will be essential also in these domains - Despite the differences among countries regarding the organization of education, the problems experienced in the evolution these professionals are shared among the professionals participating in this study - Evidence-based teaching is definitively growing to be the reference model in the near future |
| **Professionals - Communication with peers** | - Electronic communication promoted networking and improved interaction and cooperation. - In contrast, journals of doubtful quality and with less rigorous screening reviews are proliferate |
| **Professionals - Communication with the media** | - Communication with the media is currently a critical issue. The public is more and more conscious about R&D impacts in particular in the human health domains. Nevertheless, the pressure to produce news creates communication errors difficult to avoid. - However, communication with the public can bypass the media, using new communication channels (e.g. video spots on YouTube) to provide scientific content, contributing to improving the attractiveness of the profession. - There is a growing interest for science communication as an expertise area; in the last years, many research institution created new jobs for these new experts |
| **Students - Learning Critical Thinking** | - Students arrive with a set of skills very different from a few years ago. Currently many (or some) students demonstrate a mentality that they feel they are entitled to grades simply because they have watched the classes/videos or read about a certain subject. - They must have an in-depth knowledge of the concepts. Unlike morphological sciences, Physiology requires systemic, integrative thinking that should be cultivated from the first contact of the student with this area. This will allow the students to come up with new ideas and develop their experiences. - Lifelong learning is something that must be developed and nurtured. The use of clinical cases, active teaching, and knowledge of other key areas (cellular and molecular biology) will have to be increased. |
| **Students - Tools** | - The use of IT tools in the teaching-learning process is already transversal, facilitating access to information but also to multiple tutors. Mentor-teachers can be much more effective in the integrative process of reasoning with the student. - “Mentoring” teachers can use the recording of their own classes as a way to contribute with validated content; use of clinical cases, created in partnership with health professionals, can make learning more interesting. - The use of simulators may also become more accentuated, as they have improved and become more affordable. - Knowing how to program will also be an important tool. Competences in machine-learning will become more common and will improve all related processes. - In the USA there is an increase in the diversification of students (minorities, children of emigrants), which will also force the need to customize teaching techniques |
